# Profiling and Co-expression Network Analysis of Learned Helplessness Regulated mRNAs and lncRNAs in the Mouse Hippocampus

**DOI:** 10.3389/fnmol.2017.00454

**Published:** 2018-01-11

**Authors:** Chaoqun Li, Feifei Cao, Shengli Li, Shenglin Huang, Wei Li, Nashat Abumaria

**Affiliations:** ^1^Institutes of Brain Science, Collaborative Innovation Center for Brain Science, Shanghai Medical College, Fudan University, Shanghai, China; ^2^Institutes of Biomedical Sciences, Shanghai Medical College, Fudan University, Shanghai, China; ^3^Department of Laboratory Animal Science, Shanghai Medical College, Fudan University, Shanghai, China

**Keywords:** hippocampus, learned helplessness, lncRNAs, RNA-seq, synapse

## Abstract

Although studies provide insights into the neurobiology of stress and depression, the exact molecular mechanisms underlying their pathologies remain largely unknown. Long non-coding RNA (lncRNA) has been implicated in brain functions and behavior. A potential link between lncRNA and psychiatric disorders has been proposed. However, it remains undetermined whether IncRNA regulation, in the brain, contributes to stress or depression pathologies. In this study, we used a valid animal model of depression-like symptoms; namely learned helplessness, RNA-seq, Gene Ontology and co-expression network analyses to profile the expression pattern of lncRNA and mRNA in the hippocampus of mice. We identified 6346 differentially expressed transcripts. Among them, 340 lncRNAs and 3559 protein coding mRNAs were differentially expressed in helpless mice in comparison with control and/or non-helpless mice (inescapable stress resilient mice). Gene Ontology and pathway enrichment analyses indicated that induction of helplessness altered expression of mRNAs enriched in fundamental biological functions implicated in stress/depression neurobiology such as synaptic, metabolic, cell survival and proliferation, developmental and chromatin modification functions. To explore the possible regulatory roles of the altered lncRNAs, we constructed co-expression networks composed of the lncRNAs and mRNAs. Among our differentially expressed lncRNAs, 17% showed significant correlation with genes. Functional co-expression analysis linked the identified lncRNAs to several cellular mechanisms implicated in stress/depression neurobiology. Importantly, 57% of the identified regulatory lncRNAs significantly correlated with 18 different synapse-related functions. Thus, the current study identifies for the first time distinct groups of lncRNAs regulated by induction of learned helplessness in the mouse brain. Our results suggest that lncRNA-directed regulatory mechanisms might contribute to stress-induced pathologies; in particular, to inescapable stress-induced synaptic modifications.

## Introduction

Depression is among the most devastating mental disorders. The mechanisms underlying its pathologies remain to be elucidated. In addition to the classical monoaminergic hypothesis of depression pathologies (Schildkraut, 1965), studies in experimental animals and human subjects provide new insights into other possible mechanisms underlying stress and/or depression disorders. Other hypothesized mechanisms include synaptic dysfunctions (Hajszan et al., 2009; Kang et al., 2012; Duman et al., 2016), reductions in cell proliferation and neurogenesis (Newton and Duman, 2004; Czeh and Lucassen, 2007), hormonal-balance dysfunctions (Mello et al., 2003; Pariante and Lightman, 2008), changes in brain metabolism (Detka et al., 2015; Dantzer, 2017; Serafini et al., 2017) and chromatin modification and remodeling (Bagot et al., 2014; Nestler, 2014). Unraveling the exact molecular mechanisms underlying the pathologies of stress and/or depression has been one of the major challenges hampering the discovery of new, more effective antidepressant drugs.

Advances in genomic and transcriptomic research have transformed our understanding of the molecular mechanisms underlying brain functions and behavior. Numerous classes of non-coding RNAs including small non-coding RNAs such as miRNA and long non-coding RNAs (lncRNAs) such as natural antisense transcripts and long intergenic RNAs are emerging as major regulatory factors in several biological functions/dysfunctions (Faghihi and Wahlestedt, 2009; Barry and Mattick, 2012; Sauvageau et al., 2013; Kocerha et al., 2015; Huang et al., 2017). Such advances resulted in a paradigm shift in our thinking of the mechanisms underlying stress and/or depression disorders (Kocerha et al., 2015). For example, in the prefrontal cortex of depressed patients, who committed suicide, several miRNA transcripts were found to be differentially expressed (Smalheiser et al., 2012). Another profiling study in the rat brain also show that exposure to inescapable stress changes miRNA expression (Svenningsen et al., 2016). Thus, profiling studies suggest that stress and/or depression pathologies might include regulation of short non-coding RNAs expression in the brain. Furthermore, functional studies demonstrate that several miRNA transcripts could mediate neurobehavioral mechanisms of stress and/or depression disorders. The miR124a has been shown to be upregulated in the rat brain by chronic social stress (Bahi et al., 2014). Subsequent studies revealed that upregulation of the miR124a in the hippocampus of rats is sufficient to downregulate BDNF expression and induce depression-like behavior (Bahi et al., 2014). The miR16 expression in the mouse hippocampus regulates depression-like behavior via neurogenesis-dependent mechanisms (Launay et al., 2011). Finally, pharmacological studies also show that regulation of miRNAs expression might contribute to the therapeutic action of antidepressant drugs (Baudry et al., 2010).

On the other hand, studies also implicate lncRNAs in neurobehavioral mechanisms relevant to stress and depression pathologies. Long non-coding RNA regulatory mechanisms could contribute to neurodevelopmental (Bond et al., 2009), neurogenesis (Mercer et al., 2010) and synaptic plasticity/remodeling (Maag et al., 2015) related processes. Brain-derived neurotrophic factor (BDNF) expression was shown to be controlled by conserved non-coding antisense RNA transcripts in rodents (Modarresi et al., 2012) and human (Lipovich et al., 2012) tissues and cell cultures. Furthermore, the lncRNA Gomafu was shown to regulate anxiety-like behavior in mice via epigenetic mechanisms (Spadaro et al., 2015). Thus, a potential link between lncRNA and the neurobehavioral mechanisms of stress and/or depression has been proposed (Huang et al., 2017). However, the only direct evidence of a possible regulation of lncRNA expression during depression pathologies was demonstrated by a peripheral blood profiling study, in which the expression of certain lncRNAs was found to be altered in patients with major depressive disorder (Liu et al., 2014). Profiling and functional analysis of lncRNA in brain tissue from depressed subjects (postmortem) or from animal models has not been reported. It remains, therefore, unclear whether stress and/or depression pathologies could include changes in lncRNA expression in the brain.

Here, we used an animal model of depression-like symptoms, namely learned helplessness (Seligman, 1972; Chourbaji et al., 2005) in order to check if stress-induced pathologies might involve regulation of lncRNA expression in the brain. Using RNA-seq, we compared hippocampal lncRNA and mRNA transcripts expression levels among helpless mice (referred to as LH mice), non-helpless stress resilient mice (NLH) and control mice (Ctrl). Functional analysis, pathway enrichment and co-expression network analysis were then performed to provide insights into the potential regulatory roles of the identified lncRNAs.

## Materials and Methods

### Experimental Animals and Groups Comparisons

Male C57BL/6 mice (3 months old, Jie Shi Jie Experimental Animal, Shanghai, China) were group-housed (3 per cage) with free access to food and water. Animals were housed under controlled room temperature (23°C ± 2), humidity (24% ± 4) and under a 12:12 h reversed light-dark cycle, with light onset at 8:00 p.m. Behavioral experiments and other procedures were performed during the dark phase under red dim light. Animals were handled every second day for 2 min for 10 days before experiments commenced. All experiments involving animals were approved by Fudan University Committee for Animal Care and Use (license numbers: SY2014.5.007.001 and SYXK-2014-0029).

**FIGURE 1:**
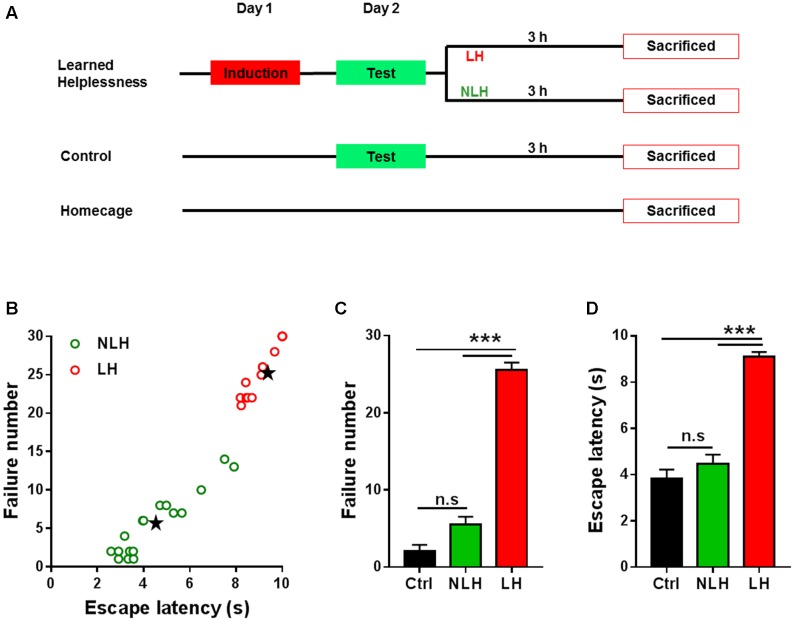
Experimental design and behavioral readouts of learned helplessness experiments. **(A)** Schematic illustration of the experimental design of learned helplessness showing three main groups of animals: learned helplessness, control and homecage. After test session on day 2 learned helplessness mice were divided into learned helpless (LH) and non-learned helpless (NLH) groups. All mice were sacrificed at the same time point; namely 3 h after test session. **(B)** Clustering analysis of mice subjected to learned helplessness procedure, using the numbers of escape failure and latency to escape (in seconds, s) as clustering parameters. Mice were classified as LH (red circles) or NLH (green circles) by clustering analysis and linear discriminant analysis (see Equations 1 and 2, Materials and Methods). Black stars represent the centroids for LH and NLH groups. **(C,D)** The numbers of escape failure **(C)** and latency to escape **(D)** during the test session on day 2 of the three groups of mice LH (*n* = 14), NLH (*n* = 17) and control (Ctrl, *n* = 10). Data are presented as mean ± SEM. One Way ANOVA was followed by Bonferroni’s *post hoc* test. n.s, non-significant; ^∗∗∗^*p* < 0.001.

Animals were randomly divided into three groups (**Figure 1A**). The learned helplessness group was exposed to inescapable foot shock (induction session) on day 1 and was tested for helplessness behavior (test session) on day 2. Following the test session, this group of animals was divided into two groups (Learned Helpless [LH] and Non-Learned Helpless [NLH] groups) based on their performance during the test session using a mathematical model (see below). The comparison between LH and NLH (referred to LH vs. NLH comparison) was used to identify learned helplessness regulated mRNA and/or lncRNA transcripts. Another group of animals was set as a control group (Ctrl), which was exposed to the test session on day 2 only without induction (i.e., on day 1 the animals were placed in shuttle box but were not exposed to inescapable foot shocks). The comparison between LH and Ctrl groups (referred to LH vs. Ctrl comparison) was also used to identify learned helplessness regulated mRNAs and lncRNAs excluding any transcripts that might be regulated in LH group by the test session *per se*. Finally, we also had a homecage group (HC) as an additional control group that did not undergo any behavioral testing, and/or handling procedure. These mice remained in their homecages throughout the experimental time course. This group was compared with Ctrl group to exclude any transcripts that might be differentially expressed by handling, placing in the shuttle box and/or testing session. All animals were sacrificed by decapitation at the same time point; namely 3 h after the test session of learned helplessness paradigm (**Figure 1A**).

### Learned Helplessness

Learned helplessness procedure was performed using the shuttle box apparatus (35 cm × 18 cm × 30 cm) connected to a computer with Graphic State 4.0 software (Coulbourn Instruments, United States). Induction and test sessions of learned helplessness in mice were performed as described before (Beurel et al., 2013). Briefly, during the induction session on day 1, the mice were exposed to 180 inescapable and unpredictable foot-shocks (0.4 mA) with various durations (1–5 s, average 3 s) and inter-shock intervals (average 17 s), amounting to total session duration of approximately 1 h. Twenty-four hours later, the test session was commenced. The mice were placed in one side of the shuttle box and left to habituate for 3 min. Following habituation phase, the guillotine door opened and a foot-shock was delivered (0.4 mA for 10 s). If the mouse successfully shuttled to the other chamber of the shuttle box, the foot-shock was terminated, door was closed, a successful escape was scored, the latency to escape was also determined and a new trial was initiated. If no shuttling was made by the animal during the 10 s foot-shock, the shock was terminated, the door was closed, a failure trial was scored, latency of 10 s was given and a new trial was initiated. In total, the mice received 30 trials of escapable foot-shocks with average inter-trial interval of 10 s (durations range, 1–24 s). The numbers of escape failure and latencies to escape were recorded automatically by the software (Graphic State 4.0 software).

Thirty-one mice were subjected to this procedure. Following test session, the mice were divided into LH and NLH subgroups (**Figure 1A**) by using *k*–means clustering analysis (*k* = 2) combined with linear regression discriminant analysis as described before (Chourbaji et al., 2005; Wang et al., 2014). Using the obtained equations (Equations 1 and 2), we were able to divide the mice into two groups; LH (*n* = 14) and NLH (*n* = 17) based on the numbers of escape failure and latency to escape (**Figure 1B**). A mouse was classified as being LH if its LH > NLH and *vice versa*. Ctrl group was not included in this analysis.

LH =−24.75+(0.23×Failure⁢ number)+(4.79×Escape⁢ latency)

NLH =−15.34+(−2.78×Failure⁢ number)+(10.31×Escape⁢ latency)

### Hippocampus Dissection and RNA Extraction

Three hours after the test session, LH (*n* = 14), NLH (*n* = 17) and Ctrl (*n* = 10) groups were sacrificed by decapitation at the same time point. HC mice (*n* = 10) were also sacrificed at the same time point with the other groups. All the brains were quickly obtained, placed on ice and hippocampi were dissected on ice and placed immediately in liquid nitrogen. All samples were stored at -80°C until use.

For RNA isolation, both sides of hippocampus from each mouse were pooled. Tissue was homogenized and total RNA was isolated using TRIzol^®^ Reagent (Invitrogen, United States) according to the manufacturer’s instruction. RNA quality was assessed using 2100 BioAnalyzer (Agilent Technologies, United States) to ensure all samples have RIN score above 6.9. RNA quantity was measured using Multiskan Go microchip (Thermo Scientific, United States).

### RNA-Seq

One microgram RNA from each animal (4 groups, *n* = 4 mice per group) was used to construct a total RNA library using VAHTS RNA Library Prep Kit (Vazyme Biotech, China) for Illumina. Each sample was individually indexed, representing a single library per mouse. The quality of the RNA library was verified by *Qsep*_100_ DNA Fragment Analyzer (Bioptic, United States). The libraries with different indices were multiplexed and loaded on an Illumina HiSeq instrument according to manufacturer’s instructions (Illumina, United States). Sequencing was carried out using a 2 × 150 PE configuration. Image analysis and base calling were conducted by the HiSeq Control Software (HCS + OLB + GAPipeline-1.6, Illumina) on the HiSeq instrument.

### Transcriptome Assembly and Quantification

Mapping of the sequencing readouts to the mouse genome (mm10) was performed using Hierarchical Indexing for Spliced Alignment of Transcripts 2 (HISAT2, Pertea et al., 2016). Assembling of RNA sequencing readouts into transcripts was conducted using String Tie software (v1.2.3) as described before (Pertea et al., 2015). The String Tie analysis was run with “–merge” mode to generate a non-redundant set of transcripts observed in all the RNA-seq samples. Cufflinks program (Trapnell et al., 2010) was used to generate the expression level for the merged transcriptome in each sample at both gene and transcript level in FPKM. All RNA-seq data were deposited at Gene expression Omnibus (GEO, accession number GSE102965).

### Functional and Pathway Enrichment Analyses

For functional analysis of the differentially expressed mRNA transcripts, we first determined all significantly different hits (Students’ *t*-test, *p* < 0.05 was used as a cutoff). The different hits were then used to perform Gene Ontology (GO) analysis. GO analysis organizes genes into hierarchical categories and can uncover gene regulatory networks on the basis of biological processes. DAVID program^1^ was used to conduct the analysis. Briefly, the significantly different hits were uploaded, and the mouse database was selected. Following the annotation summary results, GO analysis was conducted using bioprocesses as direct GO terms. Functional annotation chart was selected as results output. Fisher’s exact test was used to select the GO annotation lists that are greater than chance level (*p* < 0.05 was used as a cutoff).

For pathway enrichment analysis, ClueGO software was used as described before (Bindea et al., 2009). Briefly, the GO annotation lists from LH vs. Ctrl and LH vs. NLH comparisons were uploaded. GO-Biological processes analysis was selected. A stringent cutoff for significantly enriched pathways was selected (*p* < 0.01), before the analysis was finally conducted.

### Co-expression Network

We constructed co-expression networks to identify interactions among genes and lncRNAs. Data were pre-processed by using the average expression value of all transcripts expressed from the same gene (both mRNA and lncRNA). The co-expression networks were built according to the normalized signal intensity of individual genes. The data were then screened for differentially expressed lncRNAs and mRNAs whose expression levels correlated (positively or negatively). For each pair of lncRNA-mRNA analyzed, we used *Pearson* correlation test to detect significant correlation. Only strongly correlated (*r*^2^ ≥ 0.9, *p* < 0.01) pairs were used to construct the network and generate visual representations. In these representations, each gene corresponds to a node and the connections in between indicate strong correlation (either positive or negative). The co-expression networks were generated by using Cytoscape software (v3.4.0).

To map *cis* and *trans* co-expression networks, we tried to identify any potential *cis* mRNAs by selecting the co-expressed mRNAs within 1000 kb upstream or downstream from each of the co-expressed lncRNAs. The co-expressed mRNAs not located on the same chromosome or those located beyond 1000 kb were considered potentially *trans*.

### lncRNA Functional Analysis

To explore the possible functions of the differentially regulated and co-expressed lncRNAs that were identified, the GO analysis was applied to indicate the functions of the co-expressed genes. Each node represents either a lncRNA or a bioprocess, and the connections in between indicate strong correlation. The co-expression functional networks of the lncRNA were generated by using Cytoscape software.

### Synthesis of cDNA and Real Time PCR

One microgram total RNA from LH, NLH and Ctrl groups (*n* = 10 per group) was reversed transcribed into cDNA using Invitrogen SuperScript^®^ Reverse Transcriptase with oligo(dT)_12-18_ primers (Invitrogen, United States). Real time PCR was performed using QuantiNova^TM^ SYBR^®^ Green PCR kit (Qiagen, Germany). Each sample was run in triplicate and two independent experiments were conducted for each transcript. The expression level of mRNA and/or lncRNA transcripts was calculated using the 2^-ΔΔCt^ method. GAPDH housekeeping gene was used as an internal control to normalize the data. Results are presented as a percentage of the corresponding control for each gene experiment. All primers used for real time PCR experiments are listed in **Table 1**.

**Table 1:** Primers used for real time PCR (5′ – 3′).

Gene	Forward	Reverse
GAPDH	AGAGTGTTTCCTCGTCCCGTA	TCGCTCCTGGAAGATGGTGAT
Gm26859	GTCAGAAACACTCTCACCACA	CTATTCCTCCAAGCACCTC
Gm16364	GTCTACATAGCCACTTCTCGG	GTCTCTGCTGTCTCTCTCCC
1700109K24Rik	TAAAACAGGACTTTGGTGAG	ATTTGTCTGCTGGTCTTGAG
Nub1	CCCGCTTGGAGTATTATTGT	ACGGAGACTTGGGTTATGAG
Prkacb	CAGCCTACCAAAAAGAAGC	GCAGAAGGAAAGAACAACAG
Rbfox2	TACGCCCAAGCGACTACA	GCCAAACTGCCCAAACAT
Cacnb4	GGGAATGGACGAGG	CCAGAGCCAATCACAGC
Rab3a	TCAGCACCGTTGGCATAG	GCATTGTCCCACGAGTAAGT
Dlg2	TTCACAAAGGCTCCACT	ATTCCATTCACCGACAA
Tcf4	CAACGGAGCGATGGGTAG	GGGTGGGTTCAAGTCAGG
Grik2	GCTTGTGGAGGATGGGAAAT	AGGAGAAGACGCCTGGGT
RP24-502E20.5	GCACTGCTGCGTCATAGA	GGGCTTGGTGGCATTTA
Gomafu (Miat)	CTCAGGGTTCCTCCACTC	TGCTACATCTGTCCTCCAA


### Statistical Analysis

R-program, SAS and GraphPad Prism 7 softwares were used for conducting statistical analyses. The linear regression discriminant analysis combined with *k*–means clustering analysis (*k* = 2) were used to separate LH from NLH mice. Behavioral data were analyzed by one-way ANOVA followed by Bonferroni’s *post hoc* test. For the library differential expression analysis and cutoff *p*-value, Students’ *t*-test was used. For Go analysis Fisher’s exact test was used. For co-expression analyses *Pearson* correlation test was used (only correlations with *r*^2^ ≥ 0.9 were considered). Real time PCR data were analyzed by using two-tailed unpaired *t*-test. All data presented as mean ± SEM. *p*-value < 0.05 was considered as significantly different except with *Pearson* test and pathway enrichment, a *p* < 0.01 was considered significant.

## Results

### Identification of Helpless vs. Non-helpless Mice

Linear discriminant analysis combined with *k*–means clustering analysis of the escape latency vs. failure number resulted in two separate populations of mice, which were defined as helpless group (LH, red circles, **Figure 1B**) and non-helpless (resilient) group (NLH, green circles, **Figure 1B**) with significantly different centroids of both clusters (Black stars, **Figure 1B**). By using equations 1 and 2, we confirmed our cluster analysis. Among 31 mice, we identified 14 mice as LH and 17 mice as NLH.

In line with our clustering analysis, the number of escape failure of the identified LH mice was significantly higher than that of NLH and Ctrl mice [Bonferroni’s *post hoc* test: *p* < 0.0001; ANOVA: *F*_(2,38)_ = 164.4, *p* < 0.0001, **Figure 1C**]. Similarly, the latency to escape of LH animals was also significantly higher than that of NLH and Ctrl [Bonferroni’s *post hoc* test: *p* < 0.0001; ANOVA, *F*_(2,38)_ = 68.3, *p* < 0.0001, **Figure 1D**]. Meanwhile, there were no significant differences between NLH and Ctrl in both behavioral readouts (**Figures 1C,D**). Thus, our analysis successfully identifies the learned helpless mice and differentiates them from the learned helplessness resilient mice.

### Transcriptome Profiling and Identification of Helplessness Regulated lncRNAs

**FIGURE 2:**
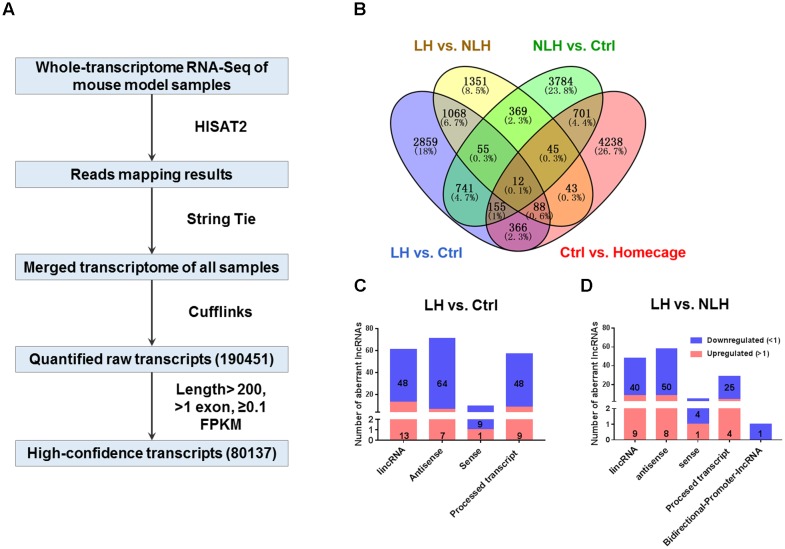
RNA-seq flow chart, profiling and classification analyses **(A)** The flow chart of RNA-seq procedure including Hierarchical Indexing for Spliced Alignment of Transcripts-2 (HISAT2), String Tie, Cufflinks and expression levels for the merged transcripts in Fragments Per Kilobase of transcript per Million mapped reads units (FPKM, see Materials and Methods) obtained from mouse hippocampal tissue (4 groups of mice, *n* = 4 mice per group). **(B)** Venn diagram showing overlap analysis to identify differentially expressed transcripts using multiple comparisons: learned helpless vs. control (LH vs. Ctrl, blue), learned helpless vs. non-learned helpless (LH vs. NLH, yellow), Ctrl vs. Homecage (red) and Ctrl vs. NLH (green). **(C,D)** The number and categories of differentially expressed lncRNAs identified in LH vs. Ctrl **(C)** and LH vs. NLH **(D)** comparisons. Blue bars represent downregulated lncRNAs, while red bars represent upregulated lncRNAs.

We performed RNA-seq on 4 mice from each group and obtained 190451 raw transcripts. After the QC step (see Methods), we narrowed down to 80137 high confidence transcripts (**Figure 2A**) for further analysis.

In order to exclude transcripts regulated by handling, contextual stimulation, testing procedure and/or transcripts related to learned helplessness, we conducted a series of comparisons among the 4 experimental groups (**Figure 2B**). First, we compared the Ctrl group with HC and found 5560 differentially expressed transcripts between both groups (**Figure 2B**). These transcripts were excluded as they might be regulated by the handling, contextual stimulation in the shuttle box and/or the test session. Second, we compared NLH with Ctrl mice and found 5821 differentially expressed transcripts (**Figure 2B**). These transcripts were more likely to be related to learned helplessness resilience phenotype.

After excluding the above described transcripts, we compared LH with Ctrl and/or NLH mice in order to identify transcripts that are specifically regulated by learned helplessness. Comparing LH with Ctrl and/or NLH resulted in identifying 3927 and 2419 transcripts (respectively, **Figure 2B**). These differentially expressed transcripts included protein coding, miscRNA, snoRNA, scaRNA, novel and pseudogene transcripts (**Tables 2**, **3**).

**Table 2:** Overview of the identified differentially expressed transcripts in LH vs. Ctrl.

	Downregulated	Upregulated	Total
Total	2550	1337	3927
lncRNAs	169	30	199
Protein coding	1340	823	2163
Novel	882	484	1366
Pseudogene	79	16	95
TEC	77	23	100
miscRNA	3	N/A	3
snoRNA	N/A	1	1


**Table 3:** Overview of the identified differentially expressed transcripts in LH vs. NLH.

	Downregulated	Upregulated	Total
Total	1663	756	2419
lncRNAs	121	21	142
Protein coding	1008	388	1396
Novel	427	320	747
Pseudogene	66	11	77
TEC	40	15	55
snoRNA	N/A	1	1
scaRNA	1	N/A	1


Importantly, we found 199 (**Table 2**) and 142 (**Table 3**) lncRNAs to be differentially expressed in LH vs. Ctrl and LH vs. NLH (respectively). Further class distribution analysis showed that LH vs. Ctrl lncRNAs included lincRNA, Antisense, sense and processed transcripts (**Figure 2C**). LH vs. NLH lncRNAs also included the same categories of lncRNA plus bidirectional promoter lncRNA transcripts (**Figure 2D**). Therefore, our results suggest that learned helplessness is associated with not only changes in mRNA expression, but also alterations in lncRNA expression. For the entire RNA-seq data and all comparisons between the above mentioned 4 groups of animals see Supplementary File 1^2^.

### Profiling, Functional and Co-expression Network Analyses of mRNA and lncRNA

In the current study, our major aims are (i) to identify differentially expressed lncRNAs in learned helpless mice and (ii) to provide insights into their possible regulatory roles based on their relationship with the identified differentially expressed mRNA transcripts.

**FIGURE 3:**
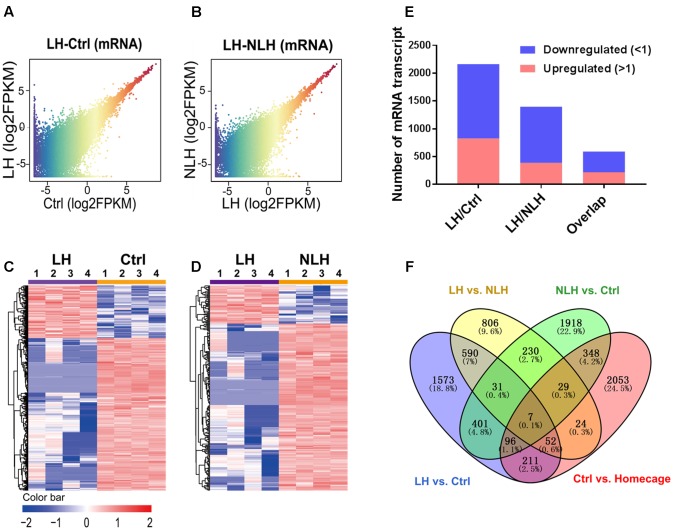
Expression and profiling of mRNA in LH vs. Ctrl and LH vs. NLH comparisons. **(A,B)** Scatter plotting of the global mRNA expression pattern in the LH vs. Ctrl **(A)** and LH vs. NLH **(B)** comparisons. **(C,D)** Heatmap hierarchical clustering analysis of differentially expressed mRNA transcripts from individual animals (*n* = 4 per group) in LH vs. Ctrl **(C)** and LH vs. NLH **(D)** comparisons. **(E)** The numbers of regulated mRNA transcripts. Blue bars represent downregulated mRNAs, while red bars represent upregulated mRNAs. **(F)** Venn diagram showing overlap analysis of differentially expressed mRNAs using multiple comparisons: learned helpless vs. control (LH vs. Ctrl, blue), learned helpless vs. non-learned helpless (LH vs. NLH, yellow), Ctrl vs. Homecage (red) and Ctrl vs. NLH (green).

To achieve these goals, we conducted profiling and functional analyses of the identified mRNA transcripts. First, we generated graphical overview of the expression signature of mRNA in LH vs. Ctrl and LH vs. NLH using scatter plotting and hierarchical clustering analysis. The scatter plotting showed that a large number of mRNAs were differentially expressed in LH group in comparison with Ctrl (**Figure 3A**) and NLH (**Figure 3B**) groups. Plotting the data using heatmap indicated that, among our differentially expressed mRNAs more were found to be downregulated in LH group in comparison with Ctrl (**Figures 3C,E**) and NLH (**Figures 3D,E**) groups. Furthermore, the number of differentially expressed mRNAs in LH vs. NLH was less than that in LH vs. Ctrl by ∼36% (LH vs. NLH 1396 mRNAs, LH vs. Ctrl 2162 mRNAs, **Figure 3F**). Thus, our results suggest that inescapable stress and/or induction of helplessness can change the expression level of a large number of protein coding mRNAs in the mouse hippocampus. Furthermore, induction of helplessness appears to predominantly downregulate gene expression among the differentially expressed mRNAs in hippocampus.

**FIGURE 4:**
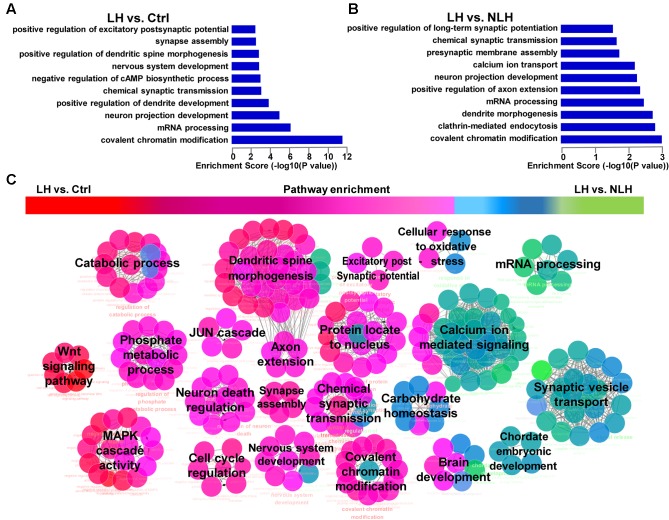
Gene Ontology and pathway enrichment analyses of mRNA. **(A,B)** The significant biological processes representing the functions of the differentially expressed mRNAs obtained from LH vs. Ctrl **(A)** and LH vs. NLH **(B)** comparisons. **(C)** Pathway enrichment analysis between LH vs. Ctrl and LH vs. NLH comparisons showing the signaling pathways that are commonly regulated in both comparisons. Red represents pathways enriched in LH vs. Ctrl comparison. Green represents pathways enriched in LH vs. NLH comparison. Pink (middle) represent signaling pathways that are commonly regulated and enriched in both comparisons.

Second, we studied whether the altered mRNAs in LH group were enriched in certain biological functions. GO analysis in LH vs. Ctrl (**Figure 4A**) and LH vs. NLH (**Figure 4B**) comparisons showed that the differentially expressed mRNAs are enriched in the following biological functions: synapse structure and function, dendritic morphology, neurodevelopment, axon and projections development, mRNA processing, calcium ion transporting and chromatin modification (**Figures 4A,B**). Next, we performed pathway enrichment analysis using Cytoscape software, to check for the signaling pathways that are commonly enriched in LH vs. Ctrl and LH vs. NLH comparisons. Enrichment analysis revealed that signaling pathways related to dendritic spine morphology, synaptic structure and function as well as chromatin modification were among the most commonly enriched signaling pathways. We also identified additional signaling pathways that were enriched, though to a lesser extent, in both comparisons (i.e., LH vs. Ctrl and LH vs. NLH) such as JUN cascade, cell cycle regulation, neurodevelopment and neuronal death regulation (**Figure 4C**). Data suggest that signaling pathways related to synapse functions and chromatin modification are among the major neuronal functions commonly regulated in both comparisons, and hence highly associated with learned helplessness behavioral phenotype.

**FIGURE 5:**
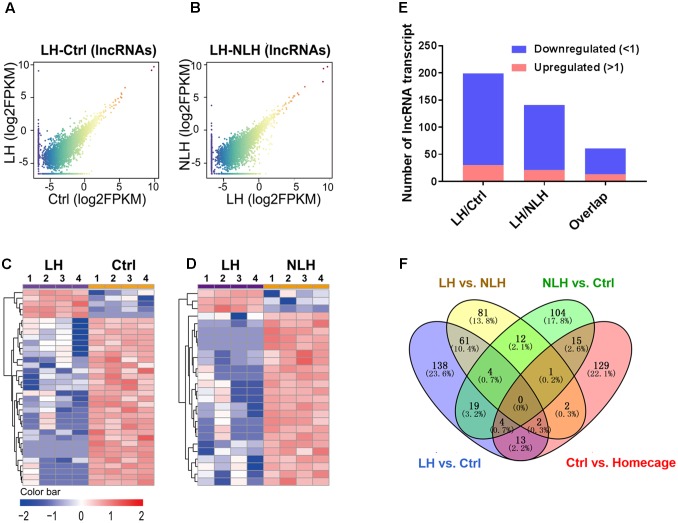
Expression and profiling of lncRNA in LH vs. Ctrl and LH vs. NLH comparisons. **(A,B)** Scatter plotting of the global lncRNA expression pattern in the LH vs. Ctrl **(A)** and LH vs. NLH **(B)** comparisons. **(C,D)** Heatmap hierarchical clustering analysis of differentially expressed lncRNA transcripts from individual animals (*n* = 4 per group) in LH vs. Ctrl **(C)** and LH vs. NLH **(D)** comparisons. **(E)** The numbers of regulated lncRNA transcripts. Blue bars represent downregulated lncRNAs, while red bars represent upregulated lncRNAs. **(F)** Venn diagram showing overlap analysis of differentially expressed lncRNAs using multiple comparisons: learned helpless vs. control (LH vs. Ctrl, blue), learned helpless vs. non-learned helpless (LH vs. NLH, yellow), Ctrl vs. Homecage (red) and Ctrl vs. NLH (green).

We proceeded with profiling and functional analyzing of the identified lncRNAs. Again, we first generated graphical overview of the expression signature of lncRNA in LH vs. Ctrl and LH vs. NLH using scatter plotting and hierarchical clustering analysis. The scatter plots showed a large number of lncRNA were found to be differentially expressed in LH group in comparison with Ctrl (**Figure 5A**) and NLH (**Figure 5B**) groups. Similar to mRNA regulation, heatmap revealed that, among the differentially expressed lncRNAs, more were found to be downregulated in LH group in comparison with Ctrl (**Figures 5C,E**) and NLH (**Figures 5D,E**) groups. Furthermore, the number of differentially expressed lncRNAs in LH vs. NLH was less than that in LH vs. Ctrl by ∼28% (LH vs. NLH 142 lncRNAs, LH vs. Ctrl 199 lncRNAs, **Figure 5F**).

**FIGURE 6:**
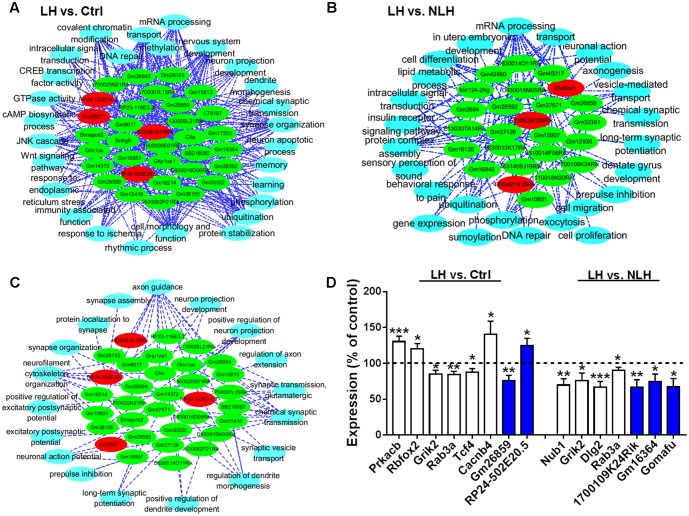
lncRNA-function co-expression network analysis and real time PCR. **(A,B)** Functional co-expression networks analysis of biological processes and the differentially expressed lncRNAs in LH vs. Ctrl **(A)** and LH vs. NLH **(B)** comparisons. **(C)** Functional co-expression network showing the synapse-related regulatory lncRNAs identified in both comparisons (LH vs. Ctrl and LH vs. NLH). Red nodes indicate upregulated lncRNAs. Green nodes indicate downregulated lncRNAs. Blue nodes indicate bioprocesses. The dotted lines indicate significant correlation (positive or negative correlation). **(D)** Quantitative analysis of selected mRNAs (white bars) and lncRNAs (blue bars) that are differentially expressed in LH vs. Ctrl (left) and LH vs. NLH (right) comparisons (*n* = 10 mice per group). Dashed line represents the control level. Data were normalized to the house keeping gene GAPDH and expressed as a percentage of the average of the corresponding control for each gene. Two-tailed unpaired *t*-test; ^∗^*p* < 0.05, ^∗∗^*p* < 0.01, ^∗∗∗^*p* < 0.001. Data are presented as mean ± SEM.

To explore the relationship between the lncRNA and mRNA transcripts, we conducted lncRNA-gene co-expression network analysis. Interestingly, we found that approximately 17% of the identified lncRNAs (34 out of 199 in LH vs. Ctrl and 24 out of 142 in LH vs. NLH) had strong and significant (*r*^2^ ≥ 0.9, *p* < 0.01, *Pearson test*) correlation with the differentially expressed mRNAs in LH vs. Ctrl (Supplementary Figure S1) and LH vs. NLH comparisons (Supplementary Figure S2). Mapping analysis of potential *cis* and *trans* mRNAs showed that none of the identified co-expressed mRNAs were located in proximity (within up to 1000 kb) to the co-expressed lncRNAs, suggesting that all the co-expressed mRNAs are potentially *trans* (Supplementary Figures S1, S2, see Supplementary Table S1 for the detailed lists of co-expression results). Next, we used these regulatory lncRNAs to conduct lncRNA-functions co-expression network analyses (see Materials and Methods) to provide insights into their possible regulatory roles. A large variety of cellular functions (representing the functions of the identified genes) correlated with the lncRNAs in LH vs. Ctrl (**Figure 6A**) and LH vs. NLH (**Figure 6B**) including synapse, chromatin modification, cell survival/differentiation related functions (**Figures 6A,B**).

Our mRNA functional analysis suggested that synapse-related genes were regulated in LH mice (**Figures 4A,B**). Pathway enrichment analyses also highlighted signaling pathways related to dendritic spine morphology, synapse assembly as well as synaptic transmission and plasticity, as major pathways commonly regulated in both comparisons LH vs. Ctrl and LH vs. NLH (**Figure 4C**). Furthermore, our lncRNA-function co-expression network analysis indicated that there is significant correlation between the regulatory lncRNAs and 18 different synapse-related functions (**Figures 6A,B**).

Such repeated pattern prompted us to focus on synapse-related lncRNAs. Hence, we conducted lncRNA-function co-expression network on lncRNAs enriched in both comparisons (LH vs. Ctrl and LH vs. NLH) with major focus on biological functions related to synapse. We found 33 out of the 58 lncRNAs (57% of the regulatory lncRNAs described above in **Figures 6A,B**) were linked to synapse-related functions such as synapse assembly, synaptic transmission, synaptic plasticity, vesicles transport, cytoskeleton and neurofilaments, spine formation and morphogenesis and protein transportation/localization to synapse (**Figure 6C**). Thus, our results predict a potential role of lncRNA-dependent mechanisms in mediating inescapable stress-induced synapse modifications.

### Quantitative Analysis of Representative mRNAs and lncRNAs

We confirmed the differential expression of a representative sample of the identified transcripts. We selected representative mRNAs and lncRNAs and quantified their expression in LH and compared it with that in Ctrl and/or NLH (in total 15 comparisons). The mRNA transcripts were selected based on their relevance to stress/depression etiologies, anxiety-/depression-like behavior in rodents and/or to synaptic functions. Prkacb was shown to be regulated by stress (Suri et al., 2014). Rbfox2 is upregulated in the brain of patients with bipolar disorders (Iwamoto et al., 2004) and mice with high anxiety-like behavior (Czibere et al., 2011). Grik2 was reported to be regulated in postmortem brain tissues obtained from depressed and suicide patients (Nagy et al., 2015). Rab3a is a synaptic protein that was shown to be suppressed in brain tissues from patients with major depressive disorder and stressed rats (Kang et al., 2012). Tcf4 is implicated in the pathologies of recurrent depressive disorders (Mossakowska-Wojcik et al., 2018). The depression etiology associated gene Cacnb4 (Rouillard et al., 2016) is known as an important regulator of synapse density (MacDonald et al., 2017). Regulation of several genes including Nub1 was reported to be associated with reversal of behavioral despair following treatments with the antidepressant drug fluoxetine (Miller et al., 2008). Dlg2 is a postsynaptic protein that was shown to be regulated in the hippocampus of depressed patients (Duric et al., 2013). Finally, the lncRNA Gomafu (also named as Miat), known to regulate anxiety-like behavior in mice (Spadaro et al., 2015), as well as other lncRNA transcripts identified in the current study including Gm26859, 1700109K24Rik, Gm16364 and RP24-502E20.5 were also quantified using real time PCR. Quantitative real time PCR analysis showed that the expression of all mRNA and lncRNA transcripts in LH mice was significantly different in comparison with the corresponding control group (**Figure 6D**). Two-tailed unpaired *t*-test results were as following: Prkacb [LH vs. Ctrl, *t*_(18)_ = 4.00, *p* = 0.0008], Rbfox2 [LH vs. Ctrl, *t*_(18)_ = 2.60, *p* = 0.018], Grik2 [LH vs. Ctrl, *t*_(18)_ = 2.80, *p* = 0.012], Rab3a [LH vs. Ctrl, *t*_(18)_ = 3.40, *p* = 0.003], Tcf4 [LH vs. Ctrl, *t*_(18)_ = 2.36, *p* = 0.029], Cacnb4 [LH vs. Ctrl, *t*_(18)_ = 2.23, *p* = 0.038], Gm26859 [LH vs. Ctrl, *t*_(18)_ = 3.23, *p* = 0.005], RP24-502E20.5 [LH vs. Ctrl, *t*_(18)_ = 2.40, *p* = 0.028], Nub1 [LH vs. NLH, *t*_(18)_ = 3.42, *p* = 0.003], Grik2 [LH vs. NLH, *t*_(18)_ = 2.32, *p* = 0.032], Dlg2 [LH vs. NLH, *t*_(18)_ = 3.98, *p* = 0.0009], Rab3a [LH vs. NLH, *t*_(18)_ = 2.22, *p* = 0.039], 1700109K24Rik [LH vs. NLH, *t*_(18)_ = 3.10, *p* = 0.006], Gm16364 [LH vs. NLH, *t*_(18)_ = 2.40, *p* = 0.027] and Gomafu [LH vs. NLH, *t*_(18)_ = 2.82, *p* = 0.011].

## Discussion

To the best of our knowledge, this is the first study to identify learned helplessness regulated lncRNAs in the brain of mouse. Using RNA-seq, we demonstrate that induction of helplessness, by exposure to inescapable stress, can misregulate not only mRNA expression, but also lncRNA expression in the mouse brain. Functional co-expression analysis predicts that some of the differentially expressed lncRNAs might play regulatory roles in several mechanisms previously implicated in stress/depression pathologies. Importantly, majority of these regulatory lncRNAs are linked to bioprocesses related to synaptic functions. Thus, our results suggest that alterations in lncRNA expression in the brain might be one of the mechanisms mediating stress-induced pathologies including the well-documented synaptic dysfunctions.

Previous profiling studies suggest that changes in the expression of distinct gene networks (coding and non-coding), in the brain, might underlie stress and depression pathologies. For example, protein-coding gene networks (mRNA) were found to be altered in the frontal cortex of depressed patients (Kang et al., 2012), hippocampus of learned helpless mice (Kohen et al., 2005) and the hippocampus of adult rats exposed to prenatal stress (Kinnunen et al., 2003). Interestingly, although the expression pattern of genes related to transcription regulation, cell survival, metabolism and developmental processes varies among these studies, majority of them show similar pattern of expression of genes related to synaptic functions. The mRNA expression signature suggests that stress and/or depression pathologies in the brain will mainly suppress gene expression including genes related synaptic vesicle trafficking, synaptic transmission and/or pre- and postsynaptic compartments (Kinnunen et al., 2003; Kohen et al., 2005; Kang et al., 2012). Furthermore, the expression of distinct non-coding gene networks (miRNA) was also found to be altered in the lateral habenula of learned helpless rats (Svenningsen et al., 2016). Pathway prediction analysis implicate the identified miRNAs in regulatory processes related to neurotrophins signaling, hormonal receptors pathways and synaptic transmission (Svenningsen et al., 2016). The currents study shows that exposure to inescapable stress and/or induction of helplessness alters the expression of not only protein coding genes (mRNAs) but also distinct groups of lncRNA networks in the mouse brain. In line with previous studies, we found that majority of the genes were suppressed by induction of helplessness. Functional and pathway enrichment analyses linked the regulated protein coding genes to bioprocesses known to be involved in stress/depression pathologies including cell survival and proliferation, neurodevelopment, cell metabolism, chromatin modifications, and most importantly synaptic functions. Moreover, we identified groups of lncRNAs that were differentially expressed in learned helpless mice in comparison with control and inescapable stress resilient mice (i.e., NLH). The results, therefore, are in line with previous profiling studies highlighting the downregulation of genes related to synaptic functions as a hallmark of stress-/depression-induced pathologies. Furthermore, the current study extends our understanding of mechanisms underlying stress-induced pathologies, beyond mRNA and miRNA, to include regulation of lncRNA expression.

Long non-coding RNAs are among the most abundant ncRNA transcripts in the brain (Derrien et al., 2012) that are believed to regulate brain functions and behavior in health and disease (Ravasi et al., 2006; Kocerha et al., 2015; Huang et al., 2017). They are hypothesized to affect brain functions by regulating epigenetic, transcriptional, splicing and/or RNA stabilizing processes (Ng et al., 2013). However, mechanisms regulating their expression and functions remain largely unknown. Thus, besides identifying distinct groups of lncRNAs related to certain behavior, it is important to also provide insights into their potential regulatory roles in the brain.

Dysfunctions in cell metabolism, cell proliferation/differentiation/survival, neurodevelopment, synapse machinery and/or epigenetic modifications are hypothesized to underlie stress and depression pathologies (Newton and Duman, 2004; Krishnan and Nestler, 2008; Bagot et al., 2014; Detka et al., 2015; Duman et al., 2016; Dantzer, 2017). Synaptic dysfunctions hypothesis is considered as one of the key hypotheses in neurobiology of stress and depression (Duman et al., 2016). Reductions in synapse density were reported in the brain of depressed patients (Kang et al., 2012) and learned helpless rats (Hajszan et al., 2009). Exposure to inescapable stress impairs synaptic transmission (Wang et al., 2014) and plasticity (Shors et al., 1989). Transcriptome profiling studies also support the synaptic dysfunctions hypothesis. Majority of synapse-related genes were found to be downregulated in brain tissue from patients with major depressive disorders (Kang et al., 2012). Similarly, exposure to prenatal stress (Kinnunen et al., 2003) or inescapable stress (Kang et al., 2012) downregulates pre-/postsynaptic gene expression in the rat brain.

Interestingly, our results are in line with the current understanding of stress/depression neurobiology. Furthermore, our analysis predicts that lncRNA-directed regulatory mechanisms might play a role in mediating stress-induced dysfunctions. Gene Ontology and pathway enrichment analysis showed that majority of the helplessness regulated mRNAs are enriched in signaling pathways implicated in stress/depression pathologies (**Figure 4**). The differentially expressed synapse-related genes were categorized as positive regulators of synaptic plasticity, transmission, assembly and morphogenesis (**Figures 4A,B**). These genes were downregulated in the hippocampus of learned helpless mice. Thus, inescapable stress could induce synaptic dysfunctions by downregulating the expression of such positive regulators, which is in line with the above described understanding of stress neurobiology. On the other hand, functional co-expression network analysis linked some groups of the differentially regulated lncRNAs to bioprocesses such as synaptic functions, cell proliferation and survival, neurodevelopment and chromatin modification (**Figures 6A,B**). These bioprocesses are implicated in the pathological mechanisms of stress and depression. Most importantly, we found that majority of the regulatory lncRNAs (57%) correlated with 18 different synapse-related functions including synaptic assembly, formation, plasticity and transmission (**Figure 6C**). Thus, our results implicate lncRNA-directed regulatory machineries in mediating stress-induced cellular dysfunctions particularly synaptic dysfunctions.

Recent advances in genomics and transcriptomics, namely the discovery of ncRNAs, triggered paradigm shift in our understanding of neurobiology of stress and depression (Kocerha et al., 2015). Identifying ncRNA networks regulated by stress is beneficial for exploring new mechanisms relevant to psychiatric disorders (Huang et al., 2017). The current study is in line with the above mentioned paradigm shift; our data implicate lncRNA-directed regulatory machineries in mediating the effects of stressful experience on brain functions and behavior. Future studies should focus on elucidating the molecular mechanisms by which lncRNAs could mediate stress effects.

## Author Contributions

CL, SH, WL, and NA conceived and designed the project, and wrote the manuscript. All experiments and data analysis were conducted by CL, FC, SL, and WL. WL and NA supervised the experiments, followed the data analysis process and monitored the data quality.

## Conflict of Interest Statement

The authors declare that the research was conducted in the absence of any commercial or financial relationships that could be construed as a potential conflict of interest.

## Footnotes

1 http://david.abcc.ncifcrf.gov

2 https://figshare.com/s/f5e3d02351ae301305a8

## Abbreviations

ANOVA: analysis of variance
Ctrl: control
Eq: equations
FPKM: Fragments Per Kilobase of transcript per Million mapped reads units
GEO: Gene Expression Omnibus
GO: Gene Ontology
HC: Homecage
HISAT2: Hierarchical Indexing for Spliced Alignment of Transcripts-2
LH: learned helplessness
lncRNA: long non-coding RNA
miRNA: microRNA
ncRNA: non-coding RNA
NLH: non-Learned Helplessness
PE: paired-end
