# Supplementary material for: Profiling and Co-expression Network Analysis of Learned Helplessness Regulated mRNAs and lncRNAs in the Mouse Hippocampus
Source: Front Mol Neurosci. 2018 Jan 11;10:454. doi: 10.3389/fnmol.2017.00454 (PMC5768633; doi:10.3389/fnmol.2017.00454)
Supplement: Supplementary file 2 [file Image_1.PDF]

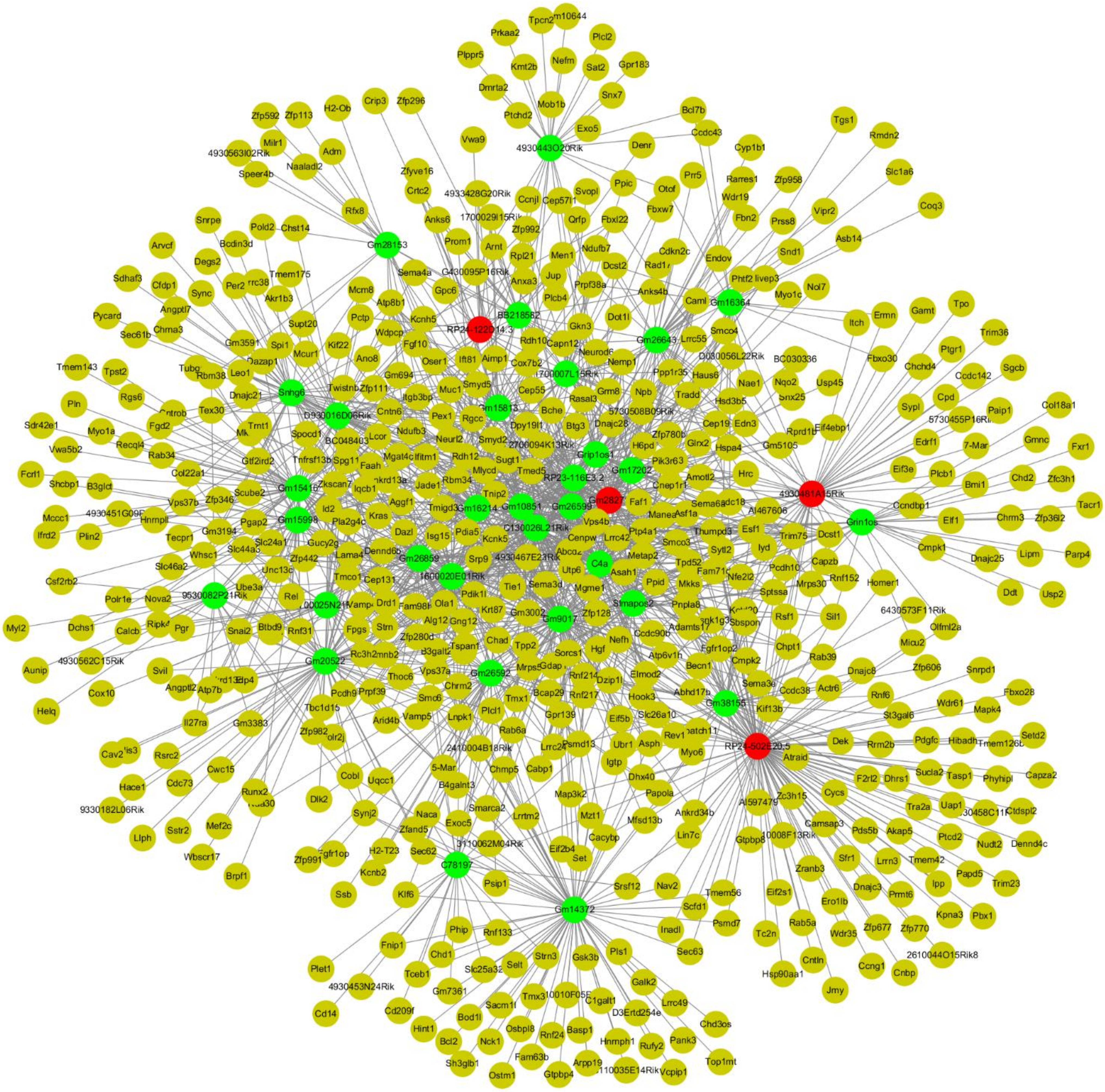

Supplementary Figure 1. Co-expression network analysis of lncRNA and mRNA in LH vs. Ctrl comparison. Red nodes represent the upregulated lncRNAs. Green nodes represent the downregulated lncRNAs. Yellow nodes represent the mRNA/genes paired with lncRNAs. The gray lines indicated a strong correlation (positive or negative) of lncRNA with mRNA. Correlations with $r^2 \geq 0.9$ and $p < 0.01$ (*Pearson* test) were used to construct the network.

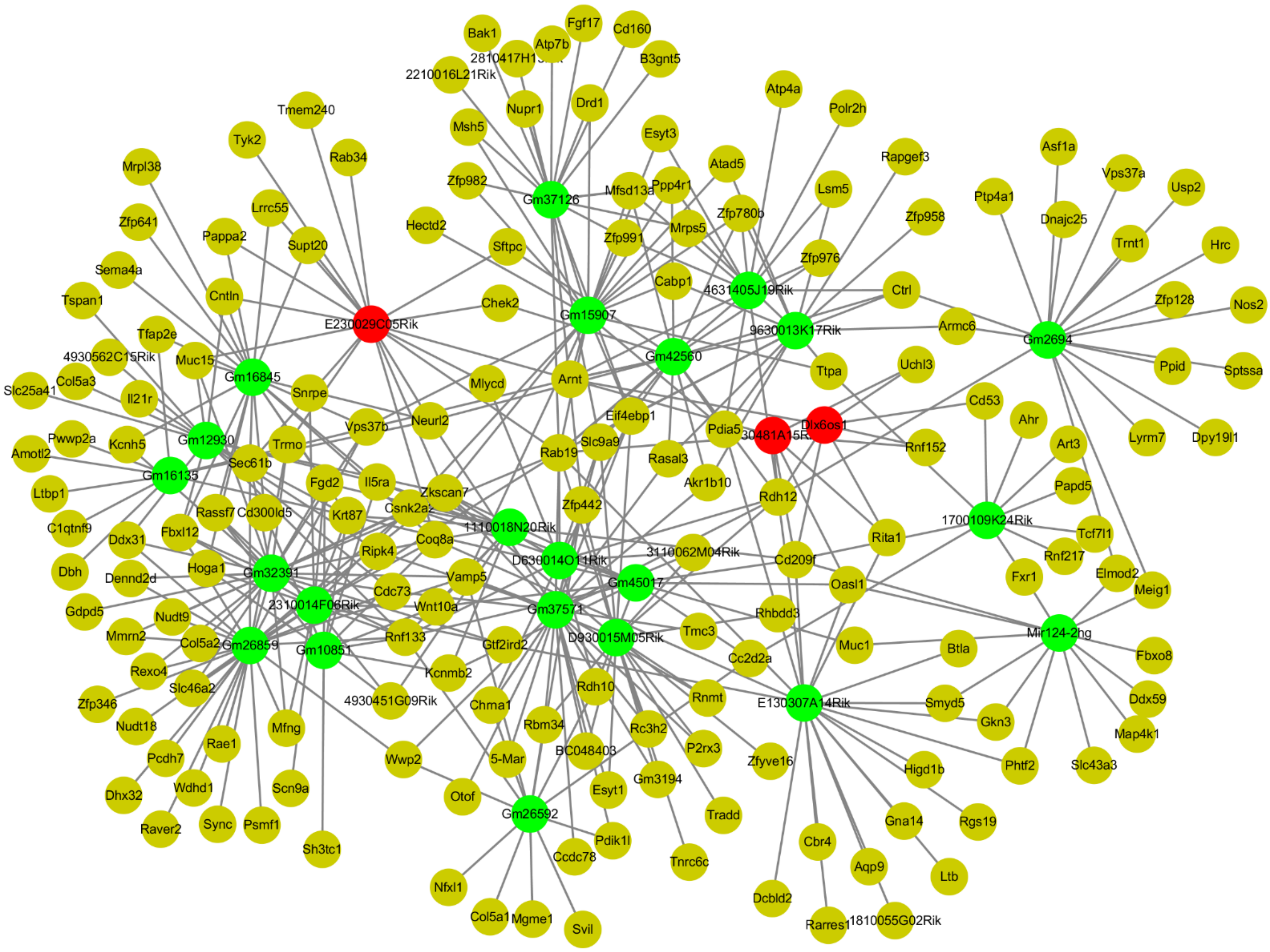

Supplementary Figure 2. Co-expression network analysis of lncRNA and mRNA in LH vs. NLH comparison. Red nodes represent the upregulated lncRNAs. Green nodes represent the downregulated lncRNAs. Yellow nodes represent the mRNA/genes paired with lncRNAs. The gray lines indicated a strong correlation (positive or negative) of lncRNA with mRNA. Correlations with $r^2 \geq 0.9$ and $p < 0.01$ (*Pearson* test) were used to construct the network.
